# Genetic diversity assessed in individuals of *Aspidosperma polyneuron* and *Cariniana estrellensis* used as seed donors in an forest gene bank

**DOI:** 10.1186/1753-6561-5-S7-P8

**Published:** 2011-09-13

**Authors:** Ana Lilia Alzate-Marin, Ronai Ferreira-Ramos, Marcela Guidugli, Carlos Alberto Martinez, Moacyr Antonio Mestriner

**Affiliations:** 1 Departamento de Genética, FMRP, Universidade de São Paulo, Ribeirão Preto, SP, Brazil; 2 Programa de Pós-Graduação em Biologia Comparada, Departamento de Biologia, FFCLRP/USP, Brazil; 3 Departamento de Biologia, FFCLRP/USP, Brazil

## Background

The neotropical tree species *Aspidosperma polyneuron* and *Cariniana estrellensis*, known as *peroba-rosa* and *jequitibá-branco* respectively, are characteristic of late secondary semideciduous Atlantic forest in submontane formation. Their wood is widely used for carpentry and construction. Increasing logging, intensive agriculture and urban expansion placed *A. polyneuron* in the Red List of Threatened Species [http://www.iucnredlist.org/apps/redlist/details/32023/0] while *C. estrellensis* is considered an endangered species. So, *in situ* conservation of these forest species is critical while *ex situ* collections as gene banks is an important complementary approach. Our study assessed and compared the genetic diversity of *A. polyneuron* and *C. estrellensis* in two stands aiming to provide subsidies for the *ex situ* conservation of these important genetic resources.

## Material and methods

Leaf tissues of mature trees of two disturbed stands (SI and SII) of *A. polyneuron* and *C. estrellensis* were sampled and stored at -20°C: SI was composed by isolated individuals of an extensive area located among Pardo river and Mogi-Guaçu river basins used as seed donor of a Forest Genetic Bank, at the University of São Paulo in Ribeirão Preto *campus* (BG-USP/RP), SP-Brazil. SII was one of the last natural populations around Ribeirão Preto region of an isolated fragment of 7.5 ha located on Águas Claras farm in Cravinhos Municipality (21°17’47’’S; 47°40’29’’W).

DNA extraction was performed according to [1]. SSR markers development for *C. estrellensis*[2] and for *A. polyneuron* (Ferreira-Ramos R., unpublished)] were used for this study (Tables 1,2). Microsatellite loci were amplified individually according [3]. FSTAT software [http://www2.unil.ch/popgen/softwares/fstat.htm] was used to calculate the genetic parameters per locus and sample: mean number of alleles (*A*), the observed (*H_o_*) and expected heterozygosities (*H_e_*). Wright’s fixation index was calculated as 1 - *H_o_/H_e_*. Deviation from Hardy-Weinberg equilibrium (HWE) was measured using GDA software[http://hydrodictyon.eeb.uconn.edu/people/plewis/software.php].

## Results and discussion

For both species, the genetic diversity was higher in SI than SII (*H_e SI/SII_*= 0.65/0.47, *H_e SI/SII_*= 0.72/0.61 for *A. polyneuron* and *C. estrellensis*, respectively). For *A. polyneuron*, the expected heterozygosity (*H_e_*) was higher than observed heterozygosity (*H_o_*) in SI and SII. For *C. estrellensis H_e_* was higher than *H_o_* in SI and the opposite was found in SII. Fixation index was higher in SI than SII for both species (F*_SI/SII_*= 0.30/0.11, F*_SI/SII_*= 0.29/-0.05 for *A. polyneuron* and *C. estrellensis*, respectively), suggesting inbreeding. Significant departures from HWE were observed for most loci in both species mainly in SI, which might be due to population substructure (Wahlund effect).

In summary, our study revealed a high diversity for the seed donor trees of *A. polyneuron* and *C. estrellensis* (SI), suggesting that this diversity was incorporated into BG-USP/RP. Indeed future progeny studies will be able to confirm these results. The low diversity found in SII for *A. polyneuron* highlights its threatened situation in this stand, on of the last natural populations in Ribeirão Preto (SP-Brazil). For *C. estrellensis*, a higher genetic diversity was observed which may be due to greater number of trees in the population, preferentially outcrossed mating system and probably gene flow from outside the fragment studied.

**Financial Support:** FAPESP, CNPq, CAPES-PROEX, FAEPA
